# Knowledge of Brucellosis, Health-Seeking Behaviour, and Risk Factors for *Brucella* Infection amongst Workers on Cattle Farms in Gauteng, South Africa

**DOI:** 10.3390/pathogens10111484

**Published:** 2021-11-14

**Authors:** Krpasha Govindasamy, Eric M. C. Etter, Bernice N. Harris, Jennifer Rossouw, Darrell A. Abernethy, Peter N. Thompson

**Affiliations:** 1 Department of Production Animal Science, Faculty of Veterinary Science, University of Pretoria, Pretoria 0110, South Africa; eric.etter@cirad.fr (E.M.C.E.); peter.thompson@up.ac.za (P.N.T.); 2 Centre de Cooperation Internationale en Recherche (CIRAD), UMR Joint Research Unit, Animals Health Territories Risks Ecosystems (ASTRE), 34070 Montpellier, France; 3 CIRAD, Institut national de la recherche agronomique (INRA), University Montpellier, 34070 Montpellier, France; 4 School of Health Systems and Public Health, University of Pretoria, Pretoria 0031, South Africa; bernice.harris@up.ac.za; 5 Centre for Emerging Zoonotic and Parasitic Diseases, National Institute for Communicable Diseases, Sandringham, Johannesburg 2192, South Africa; jennyr@nicd.ac.za; 6 Centre for Veterinary Wildlife Studies, Faculty of Veterinary Science, University of Pretoria, Pretoria 0110, South Africa; daa47@aber.ac.uk; 7 Aberystwyth School of Veterinary Science, Institute of Biological, Environmental and Rural Sciences, Aberystwyth University, Penglais, Aberystwyth SY23 3FL, UK

**Keywords:** bovine, brucellosis, human, Brucella, B. abortus, South Africa, RBT^®^, IgG ELISA^®^, IgM ELISA^®^, BrucellaCapt^®^

## Abstract

Brucellosis in humans is under-detected and underreported in sub-Saharan Africa. Risk factors associated with *Brucella* infection and health seeking behaviour in response to brucellosis-like symptoms, amongst cattle farm workers and veterinary officials in South Africa, are unknown. Farm workers and veterinary officials (*N* = 230) were screened for brucellosis using commercial Rose Bengal Test (RBT^®^), IgM Enzyme-linked Immunoassay (ELISA)^®^, IgG ELISA^®^ and the BrucellaCapt^®^ test. Knowledge of brucellosis and risk factors for exposure to *Brucella* were also investigated. Seroprevalence varied according to test used: 10.1% (RBT^®^), 20.9% (IgG ELISA^®^) and 6.5% (BrucellaCapt^®^). Only 22.2% (6/27) of veterinary officials opt to visit a clinic, doctor, or hospital in response to self-experienced brucellosis-like symptoms, compared to 74.9% (152/203) of farm workers (*p* < 0.001). Of the BrucellaCapt^®^ seropositive participants, 53% (7/15) did not visit a clinic in response to brucellosis-like symptoms. Weak evidence of an association between the handling of afterbirth or placenta and infection of a short evolution (RBT^®^, IgM ELISA^®^ and IgG ELISA^®^ seropositive) was found (OR = 8.9, 95% CI: 1.0–81.1, *p* = 0.052), and strong evidence of an association between this outcome and the slaughter of cattle (OR = 5.3, 95% CI: 1.4–19.6, *p* = 0.013). There was strong evidence of a positive association between inactive/resolved infection and veterinary officials vs. farm workers exposed to seropositive herds (OR = 7.0, 95% CI: 2.4–20.2, *p* < 0.001), with a simultaneous negative association with the handling of afterbirth or placenta (OR = 3.9, 95% CI: 1.3–11.3, *p* = 0.012). Findings suggest a proportion of undetected clinical cases of brucellosis amongst workers on cattle farms in Gauteng.

## 1. Introduction

Brucellosis is a neglected zoonotic disease of global health and economic importance [1,2,3]. Symptoms of brucellosis in humans are non-specific and are difficult to distinguish from those of other febrile illnesses [4]. They include malaise, anorexia, recurrent fever, muscular weakness, joint pain, back pain, and depression. The disease can also result in bone and testicular abscesses, endocarditis, and neurological complications and persons suffering from chronic brucellosis experience a loss of life years from persistent disability and time lost from daily activities [5].

Early detection of human brucellosis and initiation of the correct combination of antibiotics are critical for successful treatment and management of brucellosis [6] since there is no vaccine against the disease in humans [5,7]. Prevention of brucellosis in humans through public health intervention has been aimed at reducing the indirect transmission of *Brucella* bacteria through contaminated milk [8]. Reduction of infection in the animal host is usually by vaccination and test and slaughter activities conducted by governmental veterinary officials [9]. However, a lack of resource allocation to animal disease control programmes challenges the effectiveness of such programmes in low- and middle-income countries [10,11].

Poor knowledge of the zoonotic risk of brucellosis amongst people occupationally exposed to brucellosis has been identified as a further barrier to the control of livestock brucellosis and the continued spread of the disease amongst livestock [12,13]. Furthermore, such lack of knowledge is associated with ineffective precautionary behaviour needed to reduce self-exposure to contact with infected aborted material or infected cows at calving [14]. In addition, such lack of knowledge has been reported to result in inappropriate health behaviour in response to brucellosis-like symptoms which ultimately contributes to under detection and under diagnosis of brucellosis, or difficulties in medically treating clinical cases [15]. For example, self-medication for flu-like symptoms, or with an antibiotic at the suggestion of a pharmacist [6]. Other risk factors for human brucellosis have been reported to be related to the occupational status of the person [16,17], type of animal handled and the duration of contact with animals [16,17] and consumption of raw milk [18].

Culture and molecular diagnosis of brucellosis are difficult [19], therefore, use is made of serological tests to detect exposure to *Brucella* [7,20,21]. Literature supports the use of the Rose Bengal test (RBT^®^) in developing countries [22] due to the affordability, ease of conducting the test and adaptability to test serum dilutions. It can detect IgM, IgG and IgA and was found to be highly sensitive in short (“acute”) and long (“chronic”) evolution brucellosis cases when the test is optimised to have a pH capable of agglutinating blocking IgA antibodies and removing prozones. RBT^®^ was found to also be highly specific in the sera of persons with no contact with *Brucella* and its ability to detect immunoglobulin M (IgM), immunoglobulin G (IgG) and immunoglobulin A (IgA) was comparable to that of the BrucellaCapt^®^ test [22]. However, it is recommended that a complementary test be used with commercially available RBT^®^ [23] when attempting to differentiate between an infection of short or long evolution, since infections of a long evolution may be indicative of focal brucellosis or a relapse of disease. This stage is typified by an absence of IgM, an increase of IgG, IgA, and non-agglutinating antibodies [19,22].

The IgG Enzyme-linked Immunoassay (ELISA)^®^ is recognised to be a very sensitive serological test for detecting antibodies of the IgG class, which are predominately found in long evolution brucellosis cases. However, available IgG ELISA^®^ tests may have variable cut-offs due to the manufacturer differences and differences in brucellosis prevalence across areas and populations. An example of this can be found in the different cut-off reported in Hasibi et el. (2013) [24] and Peeridogaheh et al. (2013) [25]. 

The BrucellaCapt^®^, a single step immunocapture assay, has been recommended to detect relapses of brucellosis or the disease in the long evolution of infection (“chronic” stage), because of its ability to detect non-agglutinating (blocking) or incomplete antibodies, which are dominant during this stage of infection [20]. The BrucellaCapt^®^, similar to the RBT^®^, also detects IgM, IgG, and IgA antibodies. It is commercially available, both cost effective and rapid and is reported to have a sensitivity of 99.2% and a specificity of 96%, on samples determined positive by the Coomb’s test [26]. Furthermore, BrucellaCapt^®^ titres indicate the activity of infection regardless of the stage of disease, decreasing slowly after relapse and more distinctly after treatment [20]. However, BrucellaCapt^®^ was developed to diagnose brucellosis in non-endemic countries, and therefore it is still necessary to adjust the recommended cut-off titre to detect clinical cases when used in an endemic area [25,27].

There is little data on the prevalence of brucellosis in people in Africa [28]. The limited seroprevalence studies of brucellosis in humans in sub-Saharan Africa have targeted patients with fevers of unknown origin [29,30], abattoir workers [30] and the farming community and veterinarians [31,32,33,34]. However, no such study has been conducted in South Africa yet. In South Africa (SA), human brucellosis is a notifiable medical condition and bovine brucellosis is a controlled animal disease [35]. However, human brucellosis is suspected to be under detected and underdiagnosed in SA [36]. Transmission of *Brucella* to workers on farms that have brucellosis infected cattle herds is a known historical occupational hazard in the Gauteng region of SA [35,37], yet to date, no study has been conducted on cattle farms in SA to understand farm workers’ or veterinary officials’ knowledge of brucellosis, or to identify risk factors for exposure or potentially undiagnosed clinical infection, amongst these workers on farms in Gauteng.

The objectives of this study were firstly to understand knowledge of brucellosis and health seeking response to brucellosis-like symptoms amongst workers on cattle farms, and secondly to identify risk factors for *Brucella* seropositivity in this group.

## 2. Results

A total of 230 individuals were tested. Seroprevalence in this group varied depending on the test used (Table 1).

**Figure 1:**
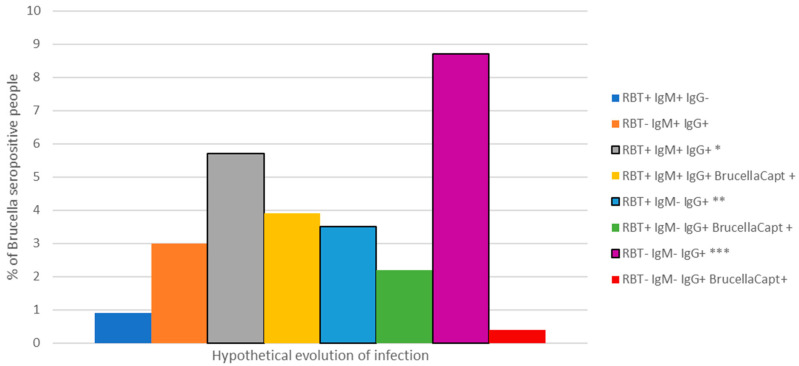
Seroprevalence and range of Brucella antibody profiles amongst study participants (*n* = 230), Gauteng, 2016. * Short evolution of infection, ** Long evolution of infection, *** Exposure or inactive/resolved infection.

Using different combinations of tests revealed a range of infection in the study group, from infection of a very short evolution (RBT^®^ and IgM ELISA^®^ positive and IgG ELISA^®^ negative) to infection of a long evolution (RBT^®^ and IgM negative and BrucellaCapt^®^ and IgG ELISA^®^ positive) (Figure 1). 

Farm workers exposed to *Brucella* infected cattle herds (30 farms), made up 65% (150/230) of the sample whilst 23% (53/230) were farm workers exposed to *Brucella* seronegative herds (11 farms). The remaining 12% (27/230) were state veterinary officials who are routinely exposed to both seropositive and seronegative *Brucella* cattle herds although not necessarily on the farms where farm workers were tested.

Using tests individually, seroprevalence ranged from 3.9% (BrucellaCapt^®^) to 16.3% (IgG ELISA^®^) amongst farm workers on case farms. On control farms, seroprevalence in farm workers ranged from 1.8% (BrucellaCapt^®^) to 5.5% (IgG ELISA^®^). Amongst veterinary officials, seroprevalence ranged from 26.6% using either the RBT^®^ or the BrucellaCapt^®^, to 74.1% (IgG ELISA^®^).

Symptoms reported by BrucellaCapt^®^ seropositive persons (*n* = 15) were distributed across titres and infection of both short and long evolution, with more symptoms being reported by those who had an antibody profile indicative of a long evolution infection (Table 2).

The majority of BrucellaCapt^®^ seropositive study participants (87%) either did not visit a clinic in response to brucellosis-like symptoms, or they attended a medical facility but were not asked their occupational history by the attending doctor (Table 3).

### 2.1. Knowledge of Brucellosis and Health Seeking Behaviour for Brucellosis-Like Symptoms

Cattle handler knowledge of brucellosis symptoms in cattle was low with 20.7% (42/203) aware that *B. abortus* can cause abortions in cattle, can cause calves to be born weak and can also be in a herd without causing abortions. Whilst 36.9% (75/203) knew that bovine brucellosis can cause disease in people, only 16.3% (33/203) reported knowing the human symptoms of disease. In contrast 63.0% (17/27) of veterinary officials knew the symptoms of bovine brucellosis and 100% knew it to be a zoonotic disease, but only 89.0% (24/27) knew the symptoms of human disease. There was a significant difference in wanting more information on brucellosis, between farm workers on case farms (OR = 2.5, 95% CI: 1.5–5.3, *p* = 0.019) and veterinary officials (OR = 7.3, 95% CI: 2.6–20.7, *p* < 0.001) vs. farm workers on control farms. Despite having greater awareness of the zoonotic nature of bovine brucellosis and human symptoms of the disease as well as wanting information more than farm workers, only 22.2% (6/27) of veterinary officials would opt to visit a clinic, doctor, or hospital in response to self-experienced brucellosis-like symptoms, compared to 74.9% (152/203) of farm workers (*p* < 0.001). We also found that 53.3% (8/15) of BrucellaCapt^®^ seropositive people did not visit a clinic in response to brucellosis-like symptoms which may result in undetected cases of brucellosis. Further findings are summarised in Table 4.

### 2.2. Univariable Analysis: Reported Brucellosis-Like Symptoms Associated with Evolution of Brucella Infection

Univariable analysis of symptoms associated with infection of short evolution (RBT^®^, IgM and IgG ELISA^®^ seropositive), long evolution (IgM ELISA^®^ seronegative and RBT^®^ and IgG ELISA^®^ seropositive) and likely inactive infection (RBT^®^ and IgM ELISA^®^ seronegative and IgG ELISA^®^ seropositive), identified a weak association between reported generalized aching and infection of short duration (OR = 4.8, 95% CI: 0.4–27.9, *p* = 0.103), and a suggestive stronger association between reported joint pain and infection of long duration (OR = 5.1, 95% CI: 0.9–33.3, *p* = 0.030). The distribution of symptoms across these stages of infection evolution and the associated significance, is shown in Table 5.

### 2.3. Univariable and Multivariable Analysis of Risk Factors Associated with Evolution of Brucella Infection

#### 2.3.1. Short Evolution (RBT^®^, IgM, IgG ELISA^®^ Seropositive)

Univariable analysis of factors associated with infection of a short evolution identified worker group, handling of afterbirth or placenta, vaccinating cattle with RB51/S19 and slaughter of cattle, for inclusion into the multivariable logistic regression model at significance *p* < 0.2 (Table 6). The handling of afterbirth or placenta was marginally significant (OR = 8.9, 95% CI: 1.0–81.1, *p* = 0.052) and slaughter of cattle significant (OR = 5.3, 95% CI: 1.4–19.6, *p* = 0.013) in the mixed effects logistic regression model fit for (RBT^®^, IgM ELISA^®^ and IgG ELISA^®^) seropositivity amongst persons tested. The random effect of clustering at farm level was not significant (*p* = 0.2137) with an intraclass correlation (ICC) of 0.16 (95% CI: 0.01–0.85).

#### 2.3.2. Long Evolution (RBT^®^, IgG ELISA^®^ Positive and IgM ELISA^®^ Negative) 

Farm workers exposed to case herds and veterinary officials compared to those exposed to seronegative herds in the exposure group variable, increasing duration of occupational exposure, and handling new-born calves were associated with infection of a long evolution (*p* < 0.02) in the univariable analysis and were included in the mixed effect multivariable logistic regression model.

In the mixed effects multivariable logistic regression model (Table 7), veterinary officials compared to farm workers exposed to seropositive herds (OR = 59.2, 95% CI: 1.0–3445.9, *p* = 0.049), was identified as marginally significant, although the small number of events of this outcome increases the uncertainty of this confidence interval. This result should therefore be interpreted with caution. The random effect of clustering at farm level was significant (LR test vs. logistic model: chibar2 = 4.68, *p* = 0.015) with an ICC of 0.51 (95% CI: 0.14–0.88). The clustering identifies that 3/5 veterinary officials in this group, were from the Germiston State Vet area. The Wald Chi2 statistic for the mixed effects model (3.88) was also marginally significant (*p* = 0.049).

#### 2.3.3. Exposure/Inactive or Resolved Infection (RBT^®^, IgM ELISA^®^ Seronegative and IgG ELISA^®^ Seropositive)

In the univariable analysis of factors associated with likely exposure/inactive or resolved infection (RBT^®^ and IgM ELISA^®^ seronegative and IgG ELISA^®^ seropositive), there was evidence of an association between the outcome and self-medicating, praying, or ignoring brucellosis-like symptoms in this group compared to those who seek out medical attention in response to symptoms. Seropositive people in this group were associated with not being engaged in the following risk activities: handling cattle at calving, handling afterbirth or placenta, handling new-born calves and milking cows. Altogether seven variables (*p* < 0.20) were identified for inclusion into the multivariable model (Table 8).

The only variable remaining associated with seropositivity in this group was veterinary officials compared to those exposed to seropositive herds in the exposure group veterinary (OR = 7.0, 95% CI: 2.4–20.2, *p* < 0.001), whilst there was strong evidence of an association between the handling of afterbirth or placenta and seronegative people in this group (OR = 3.9, 95% CI: 1.3–11.3, *p* = 0.012). This is to be expected given that the above finding that the handling of placenta and after birth was associated with people who were RBT^®^, IgM and IgG ELISA^®^ seropositive (short evolution infection) (Table 6). The random effect of clustering at farm level was non-significant and the ICC 9.04 × 10^−34^. The Wald Chi2 statistic for the mixed effects model (16.77) was significant (*p* < 0.001).

## 3. Discussion

This study identified a gap in cattle handler knowledge of brucellosis symptoms in cattle and people and identified symptoms and risk factors associated with infection of short and long evolution and likely inactive/resolved infection or exposure.

Overall cattle handler knowledge of brucellosis symptoms in cattle (29.1%) was similar to a recent global pooled awareness estimate (28.4%) for knowledge of animal symptoms of brucellosis [38], and marginally higher than the one found amongst cattle keepers (22.6%) in the Eastern Cape (E. Cape) of SA [39]. In contrast, cattle handler knowledge of brucellosis symptoms in people in this study (25%), was much lower than the global statistic (41%) [38] but higher than the one (12.7%) found in the E. Cape study [39]. Differences between the global and local proportions of awareness of human brucellosis symptoms, may be attributed to both SA studies selecting workers cattle farms. A significant source of knowledge for this group are the veterinary officials [39] whose main task is to increase cattle keepers’ knowledge of the livestock disease. The difference between cattle handler knowledge of human symptoms of brucellosis in the E. Cape study and this study, may be partially explained by greater awareness amongst veterinary officials (88.9%) who formed part of this study group as opposed to only farm workers (16.3%) in this study.

The significant difference in wanting more information on brucellosis, between farm workers on case farms, veterinary officials and farm workers on control farms was unexpected and needs to be investigated further. A possible explanation may be that veterinary officials perceived themselves to be at greater risk than farm workers or began to believe themselves to be susceptible to brucellosis. Such belief is a key construct in the health belief model of health seeking behaviour [40], triggering a drive for more information. It is also likely that exposure to the questionnaire made them realise that despite knowing brucellosis to be a zoonotic disease, they did not know the symptoms of human disease which has a direct effect on their own health, well-being, and occupational safety. 

Farm workers’ and cattle keepers’ health seeking behaviour in response to brucellosis-like symptoms also varied between provinces. In the E. Cape, 93.2% of farm workers and cattle keepers’ reported that they would go to a clinic in response to brucellosis-like symptoms experienced [39], as opposed to 68.7% in this study. The difference between farm workers and veterinary officials’ attitudes toward experiencing brucellosis-like symptoms in themselves with only 22% of veterinary officials seeking out medical care in response to brucellosis-like symptoms, is a finding of concern and needs further investigation by occupational health and safety officers.

The importance of seeking out medical care in these occupational groups is highlighted by finding that 7/15 of those that tested seropositive on the BrucellaCapt^®^ would not seek out medical care in response to brucellosis-like symptoms. It has been documented that brucellosis cases delay presenting to a medical facility from the onset of symptoms with a median delay time of 90 days [41]. Such delays increase the likelihood of complicated brucellosis, treatment failure and chronic brucellosis [6]. These findings may also suggest lack of awareness amongst medical clinicians of the occupational risk of brucellosis to farm workers and veterinary officials, which has been highlighted as a matter of concern in SA [35,36].

In this study, different risk factors were found to be associated with different serological tests combinations selected to detect infection of short and long evolution. We identified that the handling of afterbirth or placenta to be marginally significant and slaughter of cattle significantly associated with infection of a short evolution whilst infection of a long evolution was weakly associated with being a veterinary official compared to farm workers. Veterinary officials compared to farm workers were associated with inactive/resolved infection or exposure. Farm workers without this serological outcome were significantly associated with afterbirth or placenta. This is to be expected given that the above finding that the handling of placenta and after birth was associated with people who were RBT^®^, IgM and IgG ELISA^®^ seropositive (short evolution infection). Negligible clustering at farm level was evident amongst exposed or inactive/resolved infection cases, with slight clustering amongst short evolution cases and greatest clustering amongst the long evolution of infection cases. This effect can be partially explained by the presence of different categories of occupationally exposed persons in this sample. This also contributed to separation in the data, wide confidence intervals and large odds ratios which were compounded by the small number of events of seropositivity. These limitations are not uncommon and has been reported and discussed in literature. For example, in a similar study conducted, Rahman et. al. (2012) caution on the interpretation of odds ratios and confidence intervals calculated from a sample not representative of occupationally exposed persons or of insufficient size [16].

Despite this problem, these findings may be suggesting that those farm workers engaged in the slaughter of cattle were more recently exposed as opposed to those who routinely handle afterbirth or placenta. Alternatively, for slaughter of cattle there could be a recall bias as people refer to the last months rather to the last years and therefore it may not appear to be a risk factor for IgG seropositivity. Regardless, this finding indicates the importance of selecting and using appropriate screening tests in Gauteng, to determine the seroprevalence of *Brucella* amongst farm workers in bovine brucellosis endemic areas.

Veterinary officials are more regularly and frequently exposed to RB51 and S19 vaccination, as this is a fundamental bovine brucellosis control activity. Accidental exposure to RB51 through needlestick injury has been implicated as one of the main causes of brucellosis in veterinarians and their assistants [42,43]. Occupational risk to abattoir workers [33] and veterinarians has been well documented [28,44,45,46,47,48]. In this study, all the veterinary officials that tested seropositive were para-veterinarians, also known as animal health technicians, employed by the Government to perform selected veterinary services. Transmission of *Brucella* at the cattle-human-interface to officials in this context can occur through accidental self-inoculation whilst vaccinating cattle with S19 or RB51 vaccine, both of which are attenuated strains of *B. abortus* [49]. It may also occur during the collection of blood or milk samples for routine regulatory herd testing from farms participating in the provincial state veterinary services’ bovine brucellosis control programme between 2014 and 2016. Furthermore, at least 50% of AHTs reported assisting cattle with [50] dystocia, which may present a further route of transmission and exposure. Further investigation is needed to determine and mitigate the role of these variables in AHT exposure to *Brucella* on cattle farms.

The presence of significant risk factors and symptoms associated with infection of short and long evolution and poor health seeking behaviour in response to brucellosis-like symptoms among farm workers and veterinary officials with these antibody profiles, strongly suggest the presence of undetected cases of human brucellosis on cattle farms.

## 4. Materials and Methods

### 4.1. Ethical Considerations

Ethical approval for the study was granted by the Research Ethics Committee, Faculty of Health Sciences, University of Pretoria (74/2015) and the Animal Ethics committee of the University of Pretoria (V011-16). All persons were informed about the objectives of the study and counselled prior to consent on the significance of a positive test result by the medical doctor on the team. All participants were telephonically informed of their result. Reactors were revisited and further counselled on the interpretation and implication of their seropositive result. Each reactor was also given a referral letter for their doctor’s attention. This letter gave background on the study, a brief review of brucellosis and suggestions for follow-up, confirmation of disease and management of brucellosis patients to ensure the doctor was sufficiently capacitated to manage the patient.

### 4.2. Study Area and Participants

The study was conducted in Gauteng, the smallest of South Africa’s nine provinces with an area of 18,176 km^2^. In total, 41 cattle herds, participating in the Provincial Veterinary Services voluntary bovine brucellosis control programme between 2014–2016, were selected for the study using a non-probabilistic sampling strategy [51]. Of these, 30 met the definition of a case farm: “a herd with two or more cattle testing seropositive on the Rose Bengal test (RBT^®^) and the complement fixation test (CFT) at a reaction greater than or equal to 60 IU/mL”. These herds were prioritized and purposively selected to increase the probability of detecting recent exposure to *Brucella* amongst workers on cattle farms. Eleven herds were classified as control herds: “a cattle herd with a laboratory-confirmed seronegative test between 2014–2016 and no history of a seropositive herd test during 1990 to 2014”. Verification of case and control classifications was performed by cross-checking case herd records, reported by the State Veterinarians, with the Provincial Veterinary Services’ Animal Health directorate in the annual Animal Health reports.

Selection criteria for eligibility to participate in this study was occupational contact with these cattle herds.

### 4.3. Data and Sample Collection

A multidisciplinary team comprising a veterinarian, medical doctor and animal health technician visited each farm. The animal health technician served as the translator, if and when needed, and was therefore, pre-trained on the administration of the questionnaire. The veterinarian administered the questionnaire whilst the medical doctor collected blood samples from the study participants. The sampling of veterinary officials took place at the veterinary offices on appointed days for each State Vet Area. Five millilitres of blood from each participant was drawn into two tubes: (1) clot activator without serum separation and (2) EDTA anticoagulant tube. Blood samples were transported on ice, respecting the biosecurity regulations for human samples transport, to the National Institute for Communicable Diseases, Centre for Emerging Zoonotic and Parasitic Diseases Unit by the medical doctor following the farm visit, for further processing.

### 4.4. Study Design 

This study was a pilot study designed as a cross-sectional survey of workers on the selected cattle farms. All farm workers present on a farm on the day of testing were included in the sample (*n* = 203). The study was conducted on the farm sites between March and November 2016.

In addition to the farm workers, a subset of veterinary officials (*n* = 27) was included in the sample. These officials provide different services to farmers participating in the provincial bovine brucellosis control programme. Veterinary officials participating in this study was a voluntary sample of those who routinely collect blood samples and vaccinate cattle herds, vaccinate cattle without collecting blood samples, provide advisory services to cattle farmers without performing vaccinations or testing or perform diagnostic and clinical services on individual cattle. Only those veterinary officials volunteering to participate and who were available on the allocated day for testing were included in this study.

Structured questionnaires were used to collect information on risk factors for cattle handler and veterinary officials’ exposure, knowledge and health seeking response to brucellosis-like symptoms brucellosis. The questionnaire was piloted on farm workers on two farms and questions clarified from feedback gained during the pilot. Participants in the pilot study were included in the sample. All farm workers were screened on the farms using commercially available kits from Vircell Granada Spain, for the RBT^®^ [23], IgG ELISA^®^ [52,53], BrucellaCapt^®^ [54,55] and IgM ELISA^®^ [56] according to the manufacturers’ instructions and results were interpreted according to the kit guidelines. Reported sensitivity and specificity for each test is shown below (Table 9).

For the RBT^®^ test, all reagents were brought to room temperature and the antigen suspension carefully shaken. A total of 40 μL of sample, 40 μL of the positive and negative control were dispensed onto the individual circles of the test kit cards. One drop of the Rose Bengal-stained *Brucella* suspension was added close to the sample or control being analysed. The kit provided 5 mL of an acid-suspension of inactivated *Brucella abortus* antigen stained with Rose Bengal, containing phenol (concentration < 1%). Both drops were mixed until all circle surfaces were covered. The card was carefully shaken for 4 min, followed by reading of the wells for the presence or absence of agglutination.

For the IgG ELISA^®^, 100 μL of serum diluent was added to each well. A total of 5 μL of each sample, 5 μL of positive and 5 μL negative controls, with optical density (O.D.) of positive and negative controls being >0.9 and <0.55 respectively, and 5 μL of cut off control was added to the corresponding wells and shaken on a plate shaker for 2 min. The plate was then incubated for 45 min at 37 ± 1 °C for 30 min, after which excess liquid was aspirated from all wells and the wells washed 5 times with 0.3 mL of washing solution per well. Remaining liquid was drained away and 100 μL of substrate solution immediately added into each well, after which the plate was incubated at room temperature for 20 min. After this period, 50 μL of stopping solution was added into all wells. Spectrophotometer readings at 450/620 nm were taken within 1 h of stopping. The mean O.D. for the cut off control was [(<0.7 × (positive control O.D.) + >1.5 × (negative control O.D.))/2]. The antibody index was calculated as [(sample O.D./cut off serum mean O.D.) × 10]. Samples were classified as negative, equivocal, or positive if the antibody index was <9, 9–11, and >11, respectively. The IgM ELISA^®^ was conducted, and results interpreted in a similar manner as the IgG ELISA^®^, except for the initial preparation of the wells, which required 25 μL of human IgG sorbent to be added to each well to remove excess IgG antibodies or rheumatoid factor.

The BrucellaCapt^®^ test was carried out as follows: all reagents were brought to room temperature before use. A total of 50 μL of serum diluent was added into Well A, after which 50 μL of serum diluent was added into all wells (A–H). A total of 5 μL of each serum, negative and positive control were added to Well A. Doubling dilutions with 50 μL of each well was made from A to H. A total of 50 μL of the provided bacterial suspension (well homogenized by prior vigorous shaking) was added into all wells. Wells were sealed with adherent tape and incubated for 24 h at 37 °C in a chamber. Titre results were read after this and interpreted as follows: Row A—1:40, Row B—1:80, Row C—1:160, Row D—1:320, Row E—1:640, Row F—1:1280, Row G—1:2560, Row H—1:5120. Due to the paucity of information on the prevalence of endemic brucellosis in people in South Africa [51], the test was used as recommended and no adaptation made to interpret titres.

Subjects with insufficient blood for the RBT^®^ (*n* = 2) were excluded from the analysis. All samples were tested with the RBT^®^, IgG ELISA^®^ and BrucellaCapt^®^ tests. Samples that were seropositive on the ELISA IgG^®^ were tested further using the IgM ELISA^®^. Samples seronegative on the IgG ELISA^®^, but seropositive using the RBT^®,^ were also subjected to an IgM ELISA^®^ test. This selective testing of samples using the IgM ELISA^®^ was due a limited budget. The purpose was to detect the presence or absence of *Brucella* IgM antibodies in these selected samples to better understand the evolutionary stage of infection in the farm workers and veterinary officials. Stages of infection were considered along a continuum from a short evolution of infection (IgM seropositive, IgG seronegative) to a long evolution of infection (IgM seronegative, IgG seropositive, possible presence of blocking or non-agglutinating antibodies). As such, each seropositive person fell into one of five mutually exclusive groups depending on the outcome of a combination of tests: (i) RBT^®^, IgM ELISA^®^ positive and IgG ELISA^®^ negative, (ii) RBT^®^ negative and IgM, IgG ELISA^®^ positive, (iii) RBT^®^, IgM ELISA^®^ positive and IgG ELISA^®^ positive, (iv) RBT^®^, IgG ELISA^®^ positive and IgM ELISA^®^ negative, and (v) RBT^®^, IgM ELISA^®^ negative and IgG ELISA^®^ positive. Seropositive reactors on the BrucellaCapt^®^ test were allocated to the group defined by the outcomes of the RBT^®^, IgM ELISA^®^ and IgG ELISA^®^.

Subjects with test results for the IgG ELISA^®^ that were classified as equivocal (*n* = 3) were removed from the analysis. Titres were determined using the BrucellaCapt^®^ test. A titre of greater or equal to 1:320, was considered positive.

The RBT^®^ and IgM ELISA^®^ were used in series to increase the specificity of RBT^®^ to detect *Brucella* IgM, as an indication of infection of a short evolution. To detect *Brucella* IgG, an indication of a possibly longer evolution, we used the IgG ELISA^®^ test. The BrucellaCapt^®^ test was used, with the recommended cut-off titre of 1:320, for the detection of possible clinical brucellosis with either a short or long evolution. All tests were performed according to the manufacturer’s guidelines.

### 4.5. Data Management and Analysis

Completed questionnaires were captured into the electronic form function of Microsoft^®^ Access^®^ (2013), for Microsoft 365, Redmond, WA, USA. Laboratory results were captured into the appropriate record, by matching the unique identifiers of the samples.

Descriptive statistics were done in Microsoft^®^ Excel^®^ for Microsoft 365, Redmond, WA, USA. Univariable analyses were conducted in STATA 14^®^ StataCorp College Station Texas 77845 United States, for outcomes (1) RBT^®^, IgM ELISA^®^ positive and IgG ELISA^®^ positive, (2) RBT^®^. IgG ELISA^®^ positive and IgM ELISA^®^ negative, and (3) RBT^®^, IgM ELISA^®^ negative and IgG ELISA^®^ positive and (4) BrucellaCapt^®^ seropositivity amongst farm workers and veterinary officials (*N* = 230).

Univariable associations between each variable and the outcomes were assessed using Fisher’s exact test. Variables with *p* < 0.20 were selected for inclusion into mixed effects multivariable logistic regression models. Farm was included as a random effect in all three models. Veterinary officials were clustered into three groups, according to the State Vet Area they serviced. Each cluster was allocated a unique number and added to the farm variable. Three separate mixed effects logistic regression models were fit to identify risk factors for increasing evolution of infection: (1) RBT^®^, IgM ELISA^®^ positive and IgG ELISA^®^ positive (short evolution), (2) RBT^®^, IgG ELISA^®^ positive and IgM ELISA^®^ negative (long evolution), and most likely inactive or resolved infection but indicative of exposure to *Brucella* spp (3) RBT^®,^ IgM ELISA^®^ negative and IgG ELISA^®^ (exposure/inactive or resolved infection). Variables with *p* > 0.05 in the models, were systematically removed by backward elimination [57,58].

## 5. Conclusions

Evidence of cattle handler exposure to *Brucella* on cattle farms participating in the bovine brucellosis control programme in Gauteng varies depending on the serological screening test used. However, when tests results were combined to illuminate the evolution of infection in this group, significant risk factors and symptoms were found to be associated with infection of short and long evolution. This, in addition to the finding of poor health seeking behaviour in response to brucellosis-like symptoms among farm workers and veterinary officials with these antibody profiles, strongly suggest the presence of undetected cases of human brucellosis on cattle farms.

Since this study was undertaken as a pilot study, our first recommendation is to establish a representative sampling frame of occupationally exposed persons to attain a sample large and representative enough to determine the true endemic seroprevalence of brucellosis in this group and to obtain a better measure of odds ratios and confidence intervals for risk factors [16]. To achieve this, we recommend that people exposed to cattle herds in Gauteng be routinely screened for brucellosis using the RBT^®^ test as described in Diaz et al. (2011) to facilitate an early detection and response to brucellosis in these occupationally exposed persons and their families [22]. In brief, RBT^®^ should be used on plain serum and, if positive, RBT^®^ on serum dilutions up to 1/32. Dilutions should be contrasted with clinical symptoms (if any). It is also recommended that medical practitioners in SA be made aware of the clinical symptoms of both short and long evolution brucellosis and the risk of brucellosis amongst persons occupationally exposed to cattle herds in Gauteng province. With greater awareness, medical practitioners can monitor the endemic seroprevalence of brucellosis to adapt cut-off points for commercially available serological tests, such as the BrucellaCapt^®^. Awareness programmes to increase knowledge of human and cattle symptoms of brucellosis are recommended to be part of the routine veterinary regulatory service to these farms. Occupational health and safety measures to protect the health of veterinary officials should be implemented and monitored.

## Figures and Tables

**Table 1:** Seroprevalence amongst cattle handlers (*N* = 230) according to the Rose Bengal Test (RBT), IgG Enzyme-linked Immunoassay (IgG ELISA) and BrucellaCapt^®^ test combinations.

	RBT^®^	IgG ELISA^®^	BrucellaCapt^®^
RBT^®^	10.1% (23/230)	-	-
IgG ELISA^®^	9.1% (21/230)	20.9% (48/230)	-
BrucellaCapt^®^	6.1% (14/230)	6.5% (15/230)	6.5% (15/230)

**Table 2:** Distribution of BrucellaCapt^®^ titres and reported symptoms (within previous 6 months) over infection evolution (*n* = 15).

Case	BrucellaCapt^®^ Titre	Short Evolution ^*^	Long Evolution ^**^	Exposure/Inactive or Resolved Infection ^***^
1	1:320	no symptoms		
2	1:320	no symptoms		
3	1:320	no symptoms		
4	1:320	joint pain		
5	1:320	fever		
6	1:320		Fatigue	
7	1:320			no symptoms
8	1:640	no symptoms		
9	1:640	no symptoms		
10	1:640		joint pain	
11	1:640		Fever	
12	1:640		Headache	
13	1:640		Fatigue	
14	1:1280	headache		
15	1:2560	fever		

^*^ RBT^®^, IgM and IgG ELISA^®^ seropositive; ^**^ RBT^®^, IgG ELISA^®^ seropositive and IgM ELISA^®^ seronegative; ^***^ IgG ELISA^®^ seropositive and RBT^®^, IgM ELISA^®^ seronegative.

**Table 3:** Possible undetected brucellosis cases amongst BrucellaCapt^®^ seropositive study participants (*n* = 15).

BrucellaCapt^®^ Titres	Self-Medicate/ Do Nothing/Pray	Visit Clinic/Private Medical Doctor/Hospital	
		No Occupational History Taken by Attending Medical Doctor	Occupational History Taken by Attending Medical Doctor
1:320	4	2	1
1:640	3	2	1
1:1280	1	0	0
1:2560	0	1	0
Total	8 (53%)	5 (33%)	2 (13%)

**Table 4:** Distribution of responses to knowledge questions, amongst workers on cattle farms in, Gauteng, 2016.

	Farm Workers Exposed to *Brucella* Seropositive Cattle Herds (*n* = 150)		Farm workers handling *Brucella* Seronegative Cattle Herds (*n* = 53)		Veterinary Officials Exposed to *Brucella* Seropositive and Seronegative Herds (*n* = 27)		Total (*N* = 230)	
	*n*	%	*n*	%	*n*	%	*n*	%
“Do you know the symptoms of brucellosis in cattle?”								
No	112	74.7	41	77.4	10	37.0	163	70.9
Yes	38	25.3	12	22.6	17	63.0	67	29.1
Verified Yes	30	20	12	22.6	17	63.0		
“Do you know the symptoms of brucellosis in people?”								
No	129	86.0	41	77.4	3	11.1	173	75.2
Yes	21	14.0	12	22.6	24	88.9	57	24.8
“Do you understand how brucellosis causes a drop in profit?”								
No	115	76.7	39	73.6	0	0.0	154	67.0
Yes	35	23.3	14	26.4	27	100.0	76	33.0
“Do you know how brucellosis can reduce your ability to work?”								
No	120	80.0	40	75.5	6	22.2	166	72.2
Yes	30	20.0	13	24.5	21	77.8	64	27.8
“Drinking raw milk is safe and healthy”								
Do not know	14	9.3	0	0.0	0	0	14	6.1
No	62	41.3	13	24.5	25	92.6	100	43.5
Yes	74	49.3	40	75.5	2	7.4	116	50.4
“Brucellosis can cause abortions in cattle”								
Do not know	45	30.0	10	18.9	0	0.0	55	23.9
No	42	28.0	26	49.1	1	3.7	69	30.0
Yes	63	42.0	17	32.1	26	96.3	106	46.1
“Brucellosis can cause calves to be born weak”								
Do not know	49	32.7	10	18.9	1	3.7	60	26.1
No	48	32.0	29	54.7	6	22.2	83	36.1
Yes	53	35.3	14	26.4	20	74.1	87	37.8
“Brucellosis can be in the herd and not cause abortions”								
Do not know	50	33.3	10	18.9	2	7.4	54	23.5
No	67	44.7	30	56.6	2	7.4	99	43.0
Yes	33	22.0	13	24.5	23	85.2	69	30.0
“Brucellosis can cause disease in people”								
Do not know	45	30.0	11	20.8	0	0.0	49	21.3
No	44	29.3	28	52.8	0	0.0	72	31.3
Yes	61	40.7	14	26.4	27	100.0	102	44.3
Health seeking response to self-experienced brucellosis-like symptoms								
Ignore symptoms, self-medicate, pray	42	28.0	9	17.0	21	77.8	72	31.3
Visit clinic, doctor, or hospital	108	72.0	44	83.0	6	22.2	158	68.7
Medical doctor asks occupational exposure history								
Yes	22	20.4	6	13.6	4	66.7	32	20.3
No	86	79.6	38	86.4	2	33.3	126	79.7
What would you do if you observe a foetus in the field?								
Report to farm manager, private or responsible state veterinarian	103	68.7	14	26.4	26	96.3	143	62.2
Dispose of the foetus and do nothing more	47	31.3	39	73.6	1	3.7	87	37.8
Would you like more information on brucellosis?								
No	95	63.3	43	81.1	10	37.0	148	64.3
Yes	55	36.7	10	18.9	17	63.0	82	35.7

**Table 5:** Univariable analysis of *Brucella* antibody expression along the evolution of infection and brucellosis-like symptoms reported by farm workers and veterinary officials within the 6 months prior to the study, Gauteng, 2016.

Symptoms within Previous 6 Months	Study Participants (*N* = 230)	Short Evolution ^*^			Long Evolution ^**^			Exposure/Inactive or Resolved Infection ^***^		
		*n*	%	*p*-Value	*n*	%	*p*-Value	*n*	%	*p*-Value
Generalized aching				0.103			1			0.212
No	220	11	5.0		8	3.6		18	8.2	
Yes	10	2	20.0		0	0		2	20.0	
Joint pain				1			0.030			1
No	170	10	5.9		3	1.8		15	8.8	
Yes	60	3	5.0		5	8.3		5	8.3	
Fever				0.466			0.357			0.755
No	191	10	5.2		8	4.2		16	8.4	
Yes	39	3	7.7		0	0		4	10.3	
Sweating				0.698			0.362			1
No	194	12	6.2		8	4.1		17	8.8	
Yes	36	1	2.8		0	0		3	8.3	
Night-Sweating				0.475			0.357			1
No	190	12	6.3		8	4.2		17	8.9	
Yes	40	1	2.5		0	0		3	7.5	
Fatigue				0.434			0.614			0.747
No	194	10	5.2		6	3.1		18	9.3	
Yes	36	3	8.3		2	5.6		2	5.6	
Headache				0.538			0.109			1
No	161	8	5.0		8	5.0		14	8.7	
Yes	69	5	7.2		0	0		6	8.7	
Anorexia				1			0.303			1
No	220	13	5.9		7	3.2		20	9.1	
Yes	10	0	0		1	10.0		0	0	
Weight loss				1			1			0.637
No	214	12	5.6		8	3.7		18	8.4	
Yes	16	1	6.3		0	0		2	12.5	

^*^ RBT^®^, IgM and IgG ELISA^®^ seropositive; ^**^ RBT^®^, IgG ELISA^®^ seropositive and IgM ELISA^®^ seronegative; ^***^ IgG ELISA^®^ seropositive and RBT^®,^ IgM ELISA^®^ seronegative.

**Table 6:** Uni- and multivariable analysis of factors associated with *Brucella* infection of a short evolution (RBT^®^, IgM ELISA^®^ and IgG ELISA^®^ seropositivity) amongst farm worker and veterinary officials in Gauteng, 2016.

Variable and Level	Study Participants (*N* = 230)	Univariable Analysis			Multivariable Analysis		
		Short Evolution		*p*-Value			
		*n*	%		Odds Ratio	95% CI	*p*-Value
Worker group				0.089			
Farm Workers exposed to *Brucella* non-reactor herds (reference)	53	1	1.9				
Farm Workers exposed to *Brucella* reactor herds	150	9	6				
Veterinary officials	27	3	11.1				
Duration of occupation				0.286			
≤2 y	63	1	1.6				
2–6 y	59	4	6.8				
>6–14 y	54	5	9.3				
>14 y	54	3	5.6				
Brucellosis-like symptoms				0.567			
None	100	7	7				
1 or more	130	6	4.6				
Health seeking behaviour				0.551			
Self-medicate/do nothing/pray	72	5	6.9				
Visit doctor/clinic	158	8	5.1				
Drink Unpasteurized milk				1			
No	111	6	5.4				
Yes	119	7	5.9				
Handle cows at calving				0.236			
No	73	2	2.7				
Yes	157	11	7				
Handle placenta or afterbirth				0.020			
No	87	1	1.1		1	–	–
Yes	143	12	8.4		8.9	1.0–81.1	0.052
Handle newborn calves				1			
No	71	4	5.6				
Yes	159	9	5.6				
Vaccinate cattle (S19/RB51)				0.151			
No	103	3	2.9				
Yes	127	10	7.9				
Milk cows				0.561			
No	144	7	4.9				
Yes	86	6	7.0				
Slaughter cattle				0.007			
No	169	5	3.0		1	–	–
Yes	61	8	13.1		5.3	1.4–19.6	0.013

**Table 7:** Uni- and multivariable analysis of factors associated with RBT^®^, IgG ELISA^®^ positive and IgM ELISA^®^ negative farm workers and veterinary officials in Gauteng, 2016.

Variable and Level	Study Participants (*N* = 230)	Univariable Analysis			Multivariable Analysis		
		Long Evolution		*p*-value			
		*n*	%		Odds Ratio	95% CI	*p*-value
Exposure Group				0.001			
Farm workers exposed to *Brucella* non-reactor herds	53	0	0		–	–	–
Farm workers exposed to *Brucella* reactor herds	150	3	2.0		1	–	–
Veterinary officials	27	5	18.5		59.2	1.0–3445.9	0.049
Duration of occupation				0.017			
≤2 y	63	0	0				
2–6 y	58	1	1.7				
>6–14 y	52	2	3.8				
>14 y	49	5	10.2				
Brucellosis-like symptoms				0.142			
None	100	1	1.0				
1 or more	130	7	5.4				
Health seeking behaviour				0.262			
Self-medicate/do nothing/pray	72	4	5.6				
Visit doctor/clinic	158	4	2.5				
Drink unpasteurized milk				0.003			
No	111	8	7.2				
Yes	119	0	0				
Handle cows at calving				0.268			
No	73	4	5.5				
Yes	157	4	2.5				
Handle placenta or afterbirth				0.481			
No	87	4	4.6				
Yes	143	4	2.8				
Handle newborn calves				0.111			
No	71	5	7.0				
Yes	159	3	1.9				
Vaccinate cattle (S19/RB51)				0.302			
No	103	2	1.9				
Yes	127	6	4.7				
Milk cows				0.713			
No	144	6	4.2				
Yes	86	2	2.3				
Slaughter cattle				0.440			
No	169	5	3.0				
Yes	61	3	4.9				

**Table 8:** Uni- and multivariable analysis of factors associated with RBT^®^, IgM ELISA^®^ seronegative and IgG ELISA^®^ seropositive reactors amongst farm workers and veterinary officials in Gauteng, 2016.

Variable and Level	Study Participants (*N* = 230)	Univariable Analysis			Multivariable Analysis		
		Exposure/Inactive or Resolved		*p*-Value			
		*n*	%		Odds Ratio	95% CI	*p*-Value
Exposure Group				< 0.001			
Farm workers exposed to *Brucella* non-reactor herds	53	0	0		–	–	–
Farm workers exposed to *Brucella* reactor herds	150	11	7.3		1	–	–
Veterinary officials	27	9	33.3		7.0	2.4–20.3	<0.001
Duration of occupation				0.005			
≤2 y	61	2	3.3				
2–6 y	56	3	5.4				
>6–14 y	49	5	10.2				
>14 y	44	10	22.7				
Brucellosis-like symptoms				0.486			
None	100	7	6.0				
1 or more	130	13	10.0				
Health seeking behaviour				0.010			
Self-medicate/do nothing/pray	72	12	16.7				
Visit doctor/clinic	158	8	5.1				
Drink unpasteurized milk				1			
No	111	10	9.0				
Yes	119	10	8.4				
Handle cows at calving				0.010			
No	73	12	16.4				
Yes	157	8	5.1				
Handle placenta or afterbirth				0.003			
No	87	14	16.1		1	–	–
Yes	143	6	4.2		0.3	0.1–0.7	0.012
Handle newborn calves				0.005			
No	71	12	16.9				
Yes	159	8	5.0				
Vaccinate cattle (S19/RB51)				0.239			
No	103	6	5.8				
Yes	127	14	11.0				
Milk cows				0.031			
No	144	17	11.8				
Yes	86	3	3.5				
Slaughter cattle				0.603			
No	169	16	9.5				
Yes	61	4	6.6				

**Table 9:** Reported sensitivity and specific of tests used [23,52,54,56].

Test Kit (Manufacturer Vircell)	Sensitivity	Specificity
RBT^®^ *	99.0%	97.6%
IgM ELISA^®^ **	89.0%	100%
IgG ELISA^®^ **	98.0%	100%
BrucellaCapt ^®^ **	95.1%	99.0%

* Measured against another commercial Rose Bengal Card Kit; ** Measured against Coomb’s test.

## Data Availability

Data are available on request from the Gauteng Department of Agriculture and Rural Development.
